# Treatment of Polarized Cystic Fibrosis Airway Cells With HGF Prevents VX-661-Rescued F508del-CFTR Destabilization Caused by Prolonged Co-exposure to VX-770

**DOI:** 10.3389/fmolb.2021.812101

**Published:** 2021-12-22

**Authors:** Ana M. Matos, Peter Jordan, Paulo Matos

**Affiliations:** ^1^ Departamento de Genética Humana, Instituto Nacional de Saúde Doutor Ricardo Jorge, Lisboa, Portugal; ^2^ BioISI-Biosystems and Integrative Sciences Institute, Faculdade de Ciências da Universidade de Lisboa, Lisboa, Portugal

**Keywords:** cystic fibrosis, CFTR modulators, VX-661 corrector, prolonged chronic treatment, HGF

## Abstract

Cystic fibrosis (CF), the most common inherited disease in Caucasians, is caused by mutations in CFTR, the most frequent of which is F508del. F508del causes ER retention and degradation of the mutant CFTR protein, but also defective channel gating and decreased half-life at the plasma membrane. Despite the recent successes with small-molecule CFTR modulator drugs, the folding-corrector/gating-potentiator drug combinations approved for CF individuals carrying F508del-CFTR have sometimes produced severe side effects. Previously, we showed that a prolonged, 15-days treatment of polarized bronchial epithelial monolayers with the VX-809+VX-770 combination resulted in epithelial dedifferentiation effects that we found were caused specifically by VX-809. Moreover, prolonged VX-770 exposure also led to the destabilization of VX-809-rescued F508del-CFTR. Notably, co-treatment with the physiological factor HGF prevented VX-809-mediated epithelial differentiation and reverted the destabilizing effect of VX-770 on VX-809-rescued CFTR. Here, we show that prolonged treatment with VX-661, a second-generation corrector developed based on VX-809 structure, does not perturb epithelial integrity of polarized bronchial epithelial monolayers. Yet, its efficacy is still affected by co-exposure to VX-770, the potentiator present in all VX-661-containing combination therapies approved in the United States and Europe for treatment of F508del-CFTR carriers. Importantly, we found that co-treatment with HGF still ameliorated the impact of VX-770 in F508del-CFTR functional rescue by VX-661, without increasing cell proliferation (Ki-67) or altering the overall expression of epithelial markers (ZO-1, E-cadherin, CK8, CK18). Our findings highlight the importance of evaluating the cellular effects of prolonged exposure to CFTR modulators and suggest that the benefits of adding HGF to current combination therapies should be further investigated.

## Introduction

Cystic fibrosis (CF) is one of the most common life-shortening inherited diseases in Caucasian populations (De Boeck, 2020). CF is a monogenic disease caused by mutations in CF transmembrane conductance regulator (*CFTR*) gene (Saint-Criq and Gray, 2017). It encodes the CFTR protein, an anion channel expressed at the apical plasma membrane (PM) of epithelial cells, responsible for the transport of chloride and bicarbonate across different epithelia (Saint-Criq and Gray, 2017; De Boeck, 2020). The most frequent mutation, F508del, is present in at least one allele of 80–85% of CF individuals worldwide and causes the protein to misfold and be prematurely degraded by the ER quality control mechanism (ERQC) (Farinha and Matos, 2016). The very few F508del-CFTR molecules that manage to escape ERQC in CF cells bare a deficiency in channel gating, and a highly decreased half-life at the PM of epithelial cells (Farinha et al., 2013; Farinha and Matos, 2016; Loureiro et al., 2019).

Clinically, CF presents as a complex multi-organ disorder, but the respiratory complications are the disease’s major cause of morbidity and premature death (De Boeck, 2020; McBennett et al., 2021). Despite significant clinical progress in the last decades, with symptomatic therapies enabling the delay of disease progression, CF individuals inevitably develop severe chronic complications, particularly in the lungs, which greatly impact their quality of life and life expectancy (Saint-Criq and Gray, 2017; McBennett et al., 2021).

More recently, several studies using high-throughput screens of small-molecule libraries have led to the identification of selective CFTR modulator compounds capable of directly targeting the molecular defects on mutant CFTR proteins (Lopes-Pacheco et al., 2021). Several of these modulator drugs are now approved for clinical use in individuals with specific CF genotypes (Meoli et al., 2021). Such is the case of Orkambi^®^, approved by Federal Drug Administration (FDA) and European Medical Agency (EMA) in 2015 for adult CF patients and in 2018 for CF children aged 2 years and older, who are homozygous for the F508del-CFTR mutation (Boyle et al., 2014). Orkambi^®^ consists in the combination of a small-molecule CFTR corrector (a drug that facilitates CFTR protein folding, processing, and trafficking to the cell surface), named Lumacaftor (also known as VX-809) and a potentiator (a drug that improves the conductance of ions through CFTR already at the PM, maintaining the channel in an open conformation), named Ivacaftor (also known as VX-770) (Lopes-Pacheco et al., 2021). Unfortunately, the clinical response to the VX-809+VX-770 combination therapy was, at best, modest (Hubert et al., 2017; McNamara et al., 2019), with frequent respiratory adverse effects (AEs) and drug intolerance reports, leading to discontinuation of the treatment in several cases (McNamara et al., 2019). This limited efficacy of combination therapy has been partially attributed to pharmacological incompatibilities between the two drugs. Chronic VX-770 exposure reduces F508del-CFTR correction by VX-809 in CF cells, whereas VX-809 reduces plasma concentration of VX-770 through the induction of cytochrome CYP3A4 activity (Cholon et al., 2014; Schneider, 2018). However, in previous work we found that prolonged (15-days) exposure to VX-809 resulted in the dedifferentiation of epithelium-like polarized cell monolayers from both lung and intestinal origin, which represent the two systems most affected by AEs in patients treated with VX-809 or the VX-809+VX-770 combination (Matos et al., 2018). Moreover, we demonstrated that the co-treatment with hepatocyte growth factor (HGF) prevented VX-809-induced epithelial dedifferentiation and significantly improved the functional rescue of F508del-CFTR by the VX-809 +VX-770 combination. Part of this improvement resulted from HGF preventing VX-770 from destabilizing VX-809-rescued F508del-CFTR at the PM, increasing the levels of rescued maturated channel (the higher molecular weight, fully glycosylated CFTR band C in immunoblots) (Moniz et al., 2013; Matos et al., 2018). More recently, a second corrector/potentiator therapy was approved for clinical use by FDA and EMA, with the commercial designations of Symdeko^®^ and Symkevi^®^, respectively (Meoli et al., 2021). These drugs are a combination of VX-770 with Tezacaftor (VX-661), a second-generation corrector developed based on VX-809 structure but exhibiting better pharmacokinetic properties and fewer AEs reported in clinical trials (Donaldson et al., 2018; Meoli et al., 2021). The VX-661+VX-770 combination demonstrated comparable therapeutic efficacy to VX-809 + VX770 in F508del-homozygous patients (Taylor-Cousar et al., 2017) but produced improved outcomes in certain CF individuals heterozygous for F508del, in comparison to treatment with VX-770 alone (Rowe et al., 2017). At present this combination is indicated in the United States and Europe for CF individuals aged 6 years and older, homozygous for the F508del mutation or heterozygous for the F508del mutation with one of several residual function mutations (Meoli et al., 2021). Another combination drug, named Trikafta™, that combines an additional corrector, VX-445 (elexacaftor), with VX-661 and VX-770, has shown improved responses in patients with the mutation F508del in at least one allele, and has been recently approved by the FDA for the treatment of CF patients aged 6 years or older (Meoli et al., 2021). We, therefore, found it important to clarify whether the prolonged exposure to the core VX-661+VX-770 combination common to all these drugs had similar epithelial dedifferentiation effects to those found for the VX-809+VX-770 combination. Moreover, we also further investigated if co-treatment with HGF could also ameliorate any potential deleterious effects of prolonged VX-661+VX-770 treatment, and whether it also improve F508del-CFTR functional rescue in polarized epithelial airway cells.

## Materials and Methods

### Cell Culture and Treatment

CFBE41o-cells stably expressing F508del-CFTR and stably co-expressing F508del-CFTR and the YFPF46L/ H148Q/I152L halide sensor were generated and cultured as previously described (Matos et al., 2018). Cell monolayers were polarized in transwell porous (1 μm) PET filter inserts (Ø 6.4 mm, from Falcon—Thermo Fisher Scientific), pre-coated with collagen IV and fibronectin (both Thermo Fisher Scientific), and cultured in medium supplemented with 5% fetal bovine serum (FBS, Thermo Fisher Scientific), until they reached a transepithelial electrical resistance (TEER) above 600 Ω, as measured with a Chopstick Electrode (STX2 from WPI). Cells were then treated for the indicated periods, with DMSO (Sigma Aldrich) or the described concentrations of recombinant human HGF (Santa Cruz Biotechnology), VX-809, VX-770, VX-661 (Selleck Chemicals), CFTR inhibitor-172 (inh-172; CFFT United States), or forskolin (Fsk; Sigma-Aldrich). All stock solutions were made 10^3^ times concentrated, dissolved in DMSO.

### CFTR Functional Assay by Halide-Sensitive YFP

CFTR activity was determined using F508del-CFTR/HS-YFP CFBE cells polarized in transwell filter inserts, as described above. Cells were treated for 15 days with the indicated compound concentrations. Cells were then washed with PBS and incubated for 30 min in PBS containing compounds for CFTR stimulation/inhibition [Fsk, or Fsk + acVX-770 (acutely added), or inh172] at the indicated concentrations. HS-YFP fluorescence decay in polarized cells was then analyzed as previously described (Matos et al., 2018). Briefly, after stimulation, inserts were transferred to a holding chamber on top of a glass slide that can be positioned on a Leica TCS-SPE confocal microscope for time-lapse analysis. Filters were assayed individually at room temperature for iodide influx by recording fluorescence continuously (500 ms/point) for 10 s (baseline) and then for 50 s after the rapid (<1 s) apical addition, through a Ø 0.5 mm steel tubing clamp, of isomolar PBS in which 137 mM Cl^−^ was replaced by I^−^ (PBSI, final NaI concentration in the well: 100 mM). After background subtraction, cell fluorescence recordings were normalized for the initial average value measured before addition of I^−^. Quantification of fluorescence decay was performed on at least 30 individual cells per filter, using ImageJ software (NIH) as previously described (Matos et al., 2018). The initial rate of fluorescence decay (QR), an indicator of the rate of halide transport by CFTR (Galietta et al., 2001), was derived by fitting the curves to exponential decay function using GraphPad 5.0.

### Immunoblotting and Immunofluorescence

Samples were analyzed by immunoblotting as previously described (Matos et al., 2018; Loureiro et al., 2019). Antibodies used for WB were: mouse anti-CFTR clone 596 (obtained through the UNC CFTR antibody distribution program sponsored by CFFT), mouse anti-α-Tubulin clone B-5-1-2 (Sigma-Aldrich), mouse-anti-E-cadherin (Transduction Laboratories), mouse-anti-CK18 (Millipore), rabbit-anti-ZO-1, mouse-anti-CK8 and rabbit-anti-Ki-67 (all from Santa Cruz Biotechnology). Primary antibodies were detected using secondary, peroxidase-conjugated antibodies (Bio-Rad) followed by ECL. For densitometric analysis of WB bands, x-rays films were digitalized and images analyzed with ImageJ software (NIH).

For immunofluorescence analysis, cells grown on filters were fixed with 4% formaldehyde, washed with PBS, permeabilized with 0.2% Triton X-100 (Sigma-Aldrich), and incubated for 1 h with mouse anti-CFTR clone 570 (obtained through the UNC CFTR antibody distribution program sponsored by CFFT). Cells were then thoroughly washed with PBS and incubated for 30 min with AlexaFluor488-conjugated secondary antibody (Life Technologies Invitrogen Corporation). Actin was stained using phalloidin-TRITC (Jackson ImmunoResearch Laboratories), followed by thorough washing in PBS and DAPI staining of nuclei. Filters were mounted on microscope slides with Vectashield (Vector Laboratories), covered with coverslips and sealed. Images were recorded on a Leica TCS-SPE confocal microscope and assembled into figures with Adobe Photoshop software.

### Statistical Analysis

Quantitative results are shown as means ± SEM of at least five replicate observations. To compare sets of data, we used either one-way or two-way ANOVA followed by Tukey’s or Bonferroni posttests, respectively, as indicated. Differences were considered significant when *p* < 0.05.

## Results and Discussion

### In Contrast to VX-809, Prolonged Treatment With VX-661 Does not Compromise Epithelial Integrity of Polarized Bronchial Epithelial Cells

We previously described that a prolonged, 15-days treatment of polarized bronchial epithelial cells with the VX-809 corrector drug led to formerly unrecognized epithelial dedifferentiation effects (Matos et al., 2018). In contrast to the commonly used 48 h *in vitro* treatments, prolonged exposure of F508del-CFTR-expressing CFBE cells to 3 µM VX-809 resulted in decreased transepithelial resistance and concomitant downregulation of epithelial differentiation markers, namely the tight junction protein Zonula occludens-1 (ZO-1) (Martin and Jiang, 2009) and the pro-differentiative marker cytokeratin 18 (CK18) (Zhang et al., 2014), whereas the lung cancer pro-dedifferentiation marker cytokeratin 8 (CK8) (Fukunaga et al., 2002) became upregulated (Matos et al., 2018).

**FIGURE 1:**
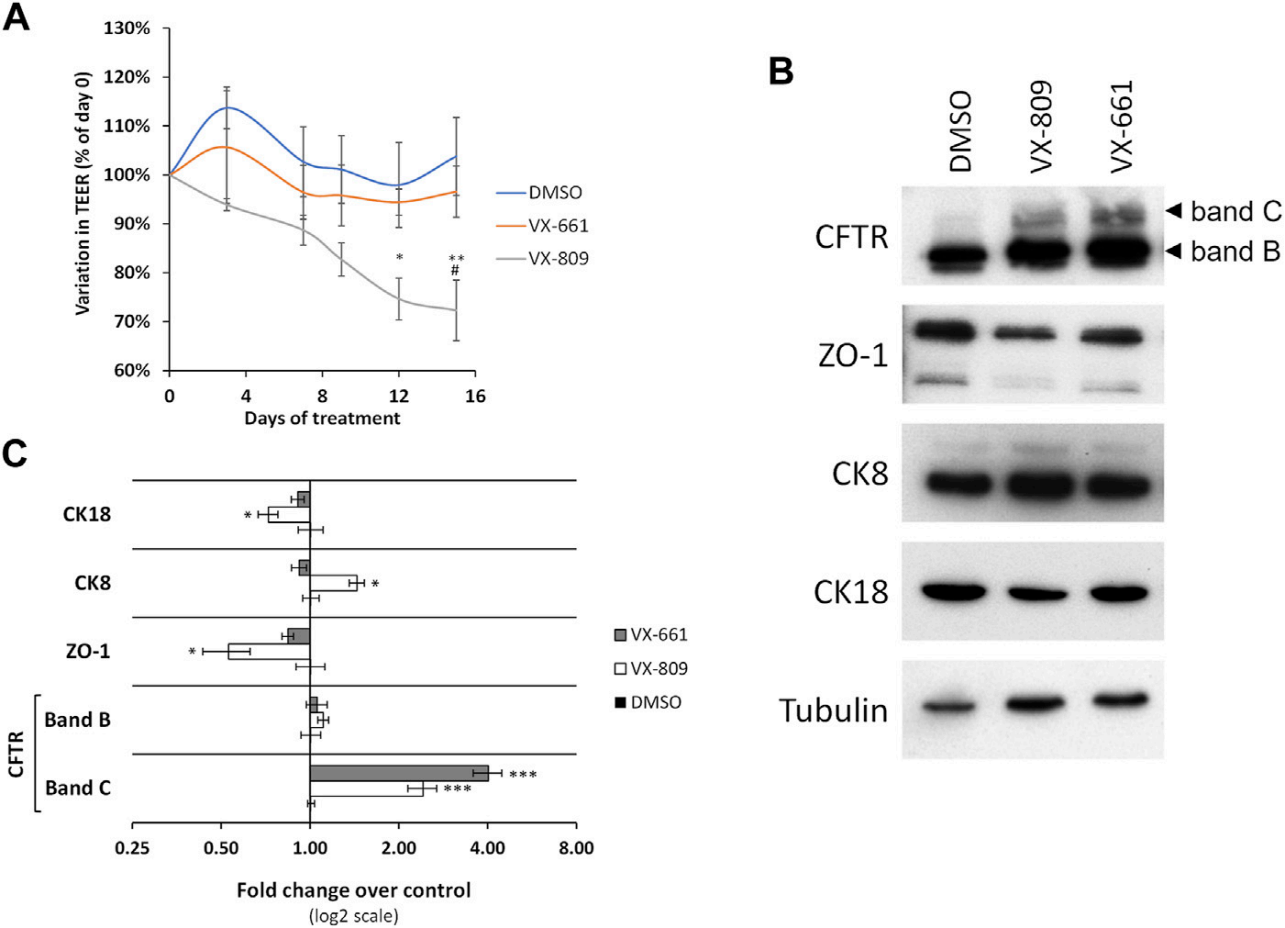
Prolonged treatment with VX-661 does not compromise epithelial integrity in polarized F508del-CFTR CFBE cells. **(A)** Variation in TEER of polarized F508del-CFTR CFBE cells treated for 15 days with vehicle (DMSO) or 3 μM of either VX-809 or VX-661. **(B)** WB analysis of whole cell lysates from polarized F508del-CFTR CFBE cells treated as in **(A)**. Shown are representative images of immunoblots using antibodies against the indicated proteins. **(C)** Bar plots of immunoblot [as in **(B)**] band intensity quantification, normalized to DMSO. Tubulin was used as a loading normalizer in band intensity quantification. Data are means ± SEM from at least five independent assays. Statistical significance was assessed using two-way ANOVA [F_treatment_ = 15.95 **(A)** and 20.28 **(C)**, both *p* < 0.0001) followed by Bonferroni posttests (*p < 0.05, **p < 0.01, and ***p < 0.001, relative to DMSO and ^#^ *p* < 0.05 relative to VX-661).

We therefore investigated whether the prolonged exposure of polarized F508del-CFBE cells to VX-661 had similar epithelial differentiation effects to VX-809. We found that in contrast to VX-809 treatment, which progressively decreased TEER reaching a significant reduction over control conditions at 12 days of treatment, TEER values for VX-661-treated cells showed no significant difference from control cells during the entire 15 days of treatment and were significantly higher than VX-809-treated cells at day 15 (Figure 1A). Moreover, whereas ZO-1 and CK18 levels were significantly decreased after 15 days in VX-809-treated cells, the levels of these epithelial markers in VX-661-treated cells remained comparable to those of control cells (Figures 1B,C). Both these observations are consistent with VX-661 having a better safety profile, with far less adverse effects, respiratory and otherwise, in clinical trials (Taylor-Cousar et al., 2017; Donaldson et al., 2018) and after continued clinical use (Gavioli et al., 2021; Paterson et al., 2021).

**FIGURE 2:**
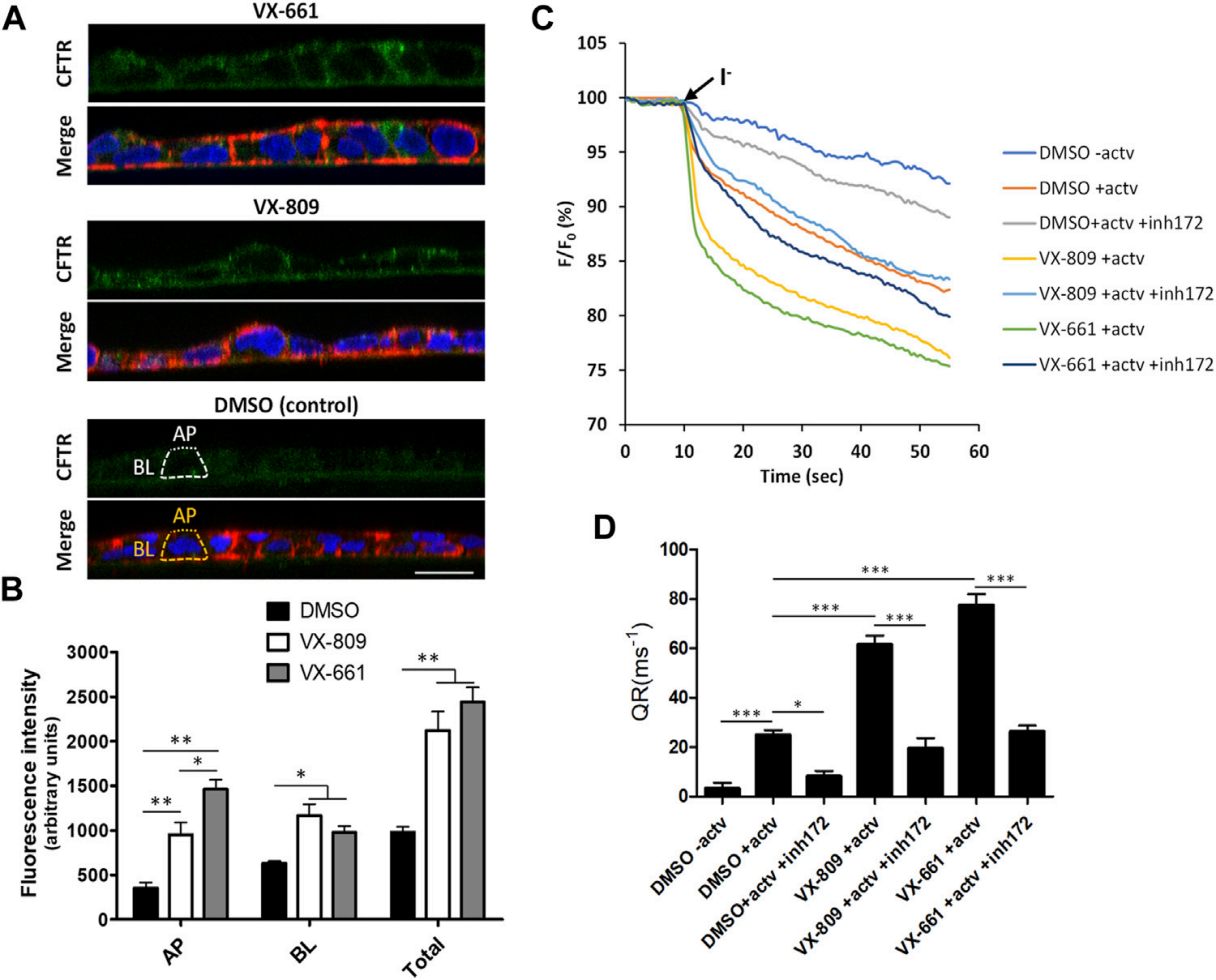
Compared to VX-809, prolonged treatment with VX-661 favors the apical localization and function of rescued F508del-CFTR. **(A)** Immunofluorescence staining of polarized F508del-CFTR CFBE cells treated as in Figure 1A. Cells were stained with anti-CFTR/Alexa 488 (green), phalloidin-TRITC (red) and DAPI (blue), and analyzed by confocal microscopy. Shown are merged images of the three color channels **(lower panels)** as well as isolated CFTR-staining **(green channel-upper panels)** representative of the indicated treatment conditions. Overlay interrupted lines exemplify the method used for CFTR signal quantification. Actin signal (yellow lines) was used as a guide to define the apical (AP—dot lines) and basolateral (BL—trace lines) membrane regions that were used to quantify CFTR signal intensity (white trace and dot lines, respectively). **(B)** Plotted are means ± SEM of AP, BL, and Total (BL + AP) signal intensities from at least 25 cells analyzed in each of three independent experiments. Two-way ANOVA identified significant variations among treatments (*F* = 23.18, *p* < 0.0001) and subcellular localizations (*F* = 169.1, *p* < 0.0001). Bonferroni posttests were used to compare treatments at the different subcellular localizations. *p < 0.05; **p < 0.01. **(C)** Representative traces of fluorescence decay on iodide influx assays of polarized HS-YFP/F508del-CFTR CFBE cells treated as in Figure 1A and stimulated, prior to the addition of iodide (I^−^), with either DMSO (− actv) or 5 μM forskolin and 10 μM VX-770 (+actv) for 30 min, in the presence or absence of 25 μM CFTR inhibitor 172 (inh172). Fluorescence was recorded continuously, first for 10 s (baseline) and then for 50 s after the rapid (≤1 s) addition of isomolar PBS, in which Cl^−^ was replaced by I^−^. Fluorescence (F) was plotted over time as percentage of fluorescence at time 0 (F_0_). Data are means ± SEM of five independent assays. **(D)** Fluorescence decay rates (QR), proportional to the initial influx of I^−^ into the cells, calculated by fitting the curves to the exponential decay function. Data are means ± SEM of five independent assays. Statistical significance between treatments was assessed using one-way ANOVA (*F* = 75.16, *p* < 0.0001) followed by Tukey’s posttests (*p < 0.05 and ***p < 0.001).

In addition, we observed that prolonged treatment with VX-661 elicited an apparent improved rescue of F508del-CFTR (rF508del) in polarized CFBE cells compared to VX-809 (Figures 1B,C). By immunolabeling polarized CFBE cells treated with either vehicle (DMSO), or 3 µM of either VX-809 or VX-661 for 15 days, we could observe that exposure to VX-661 resulted in a clearly better structured epithelial-like monolayer, when compared to VX-809, and also elicited apparent stronger CFTR staining at the apical membrane of the polarized cell monolayer (Figure 2A). Using a previously described methodology (Loureiro et al., 2019) to quantify apical (AP), basolateral (BL) and total (TL = AP + BL) immunofluorescent CFTR signals, we confirmed that, despite producing equivalent levels of total rF508del protein, prolonged treatment with VX-661 resulted in a small (∼1.5-fold) but significant (*p* < 0.05) increase in apical rF508del abundance over that produced by equivalent treatment with VX-809 (Figure 2B). Repeating these experiments using the previously characterized model of polarized CFBE cell co-expressing F508del-CFTR and the YFP-F46L/H148Q/I152L halide sensor (Matos et al., 2018; Loureiro et al., 2019) allowed us to confirm that forskolin-stimulated activity of CFTR was indeed higher in VX-661-treated cells, although not sufficient to reach statistical significance over cells similarly treated with VX-809 (Figures 2C,D). In both cases, CFTR activity was similarly inhibited by the presence of CFTR inhibitor 172 (inh172; Figures 2C,D).

## Co-Treatment with HGF Prevents Apical Levels of VX-661-Rescued F508del-CFTR From Decreasing During Chronic Exposure to VX-770 Potentiator

**FIGURE 3:**
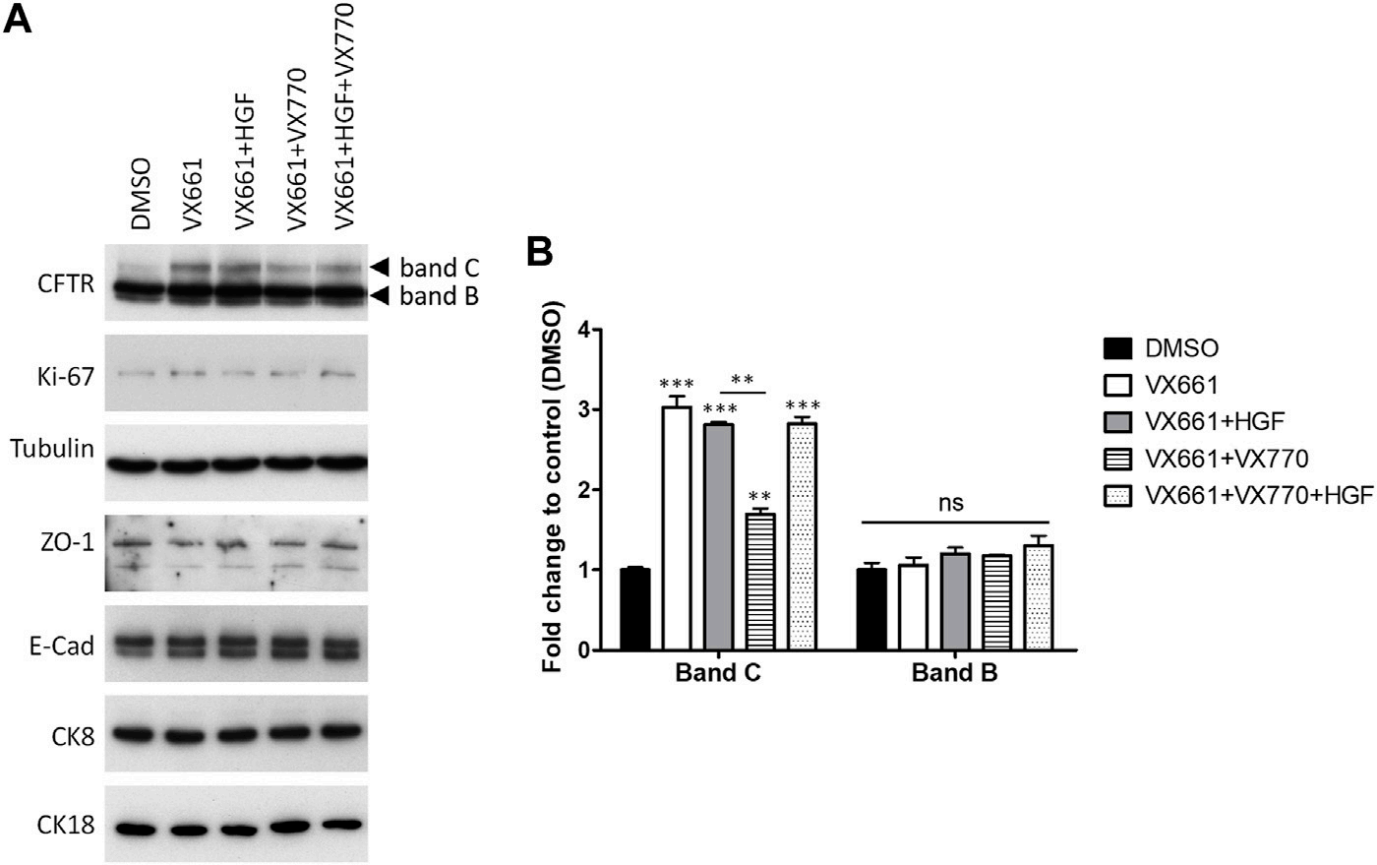
Co-treatment with HGF reverts the downregulation of VX-661-rescued F508del CFTR by prolonged exposure to VX-770. **(A)** WB analysis of whole cell lysates from polarized F508del-CFTR CFBE cells treated for 15 days with either vehicle (DMSO), 3 μM VX-661, 1 μM VX-770, 50 ng/ml HGF or the indicated combinations of these substances. Shown are representative images of immunoblots using antibodies against the indicated proteins. **(B)** Bar plots of CFTR immunoblot quantification showing the variations in CFTR band C (mature, fully glycosylated channel) and band B (immature, ER-accumulated channel) intensities, normalized to DMSO. Tubulin was used as a loading normalizer in band intensity quantification. Data are means ± SEM from at least five independent assays. Statistical significance was assessed using one-way ANOVA (F_band C_ = 119.1, *p* < 0.0001) followed by Tukey’s posttests (**p < 0.01, ***p < 0.001). Note that, as with CFTR band B, none of the other makers showed significant variations among treatments (ns).

We previously showed that co-treatment with 50 ng/ml HGF could ameliorate the differentiation effects of prolonged VX-809 exposure, also enhancing the rescue of F508del-CFTR by the corrector in polarized CFBE cells (Matos et al., 2018). Postulating that the two effects could be related, we investigated whether HGF would also enhance the activity of VX-661 in these cells. Interestingly, although we confirmed that the prolonged treatment with HGF did not alter the proliferative potential of these cells (assessed through the levels of proliferation marker Ki-67; Figure 3A), when comparing VX-661 + HGF co-treated cells to cells treated with VX-661 alone (Figures 3A,B), we observed no improvement in rF508del levels nor any significant change in the abundance of epithelial markers, including ZO-1, E-cadherin (E-cad), CK18 or CK8.

**FIGURE 4:**
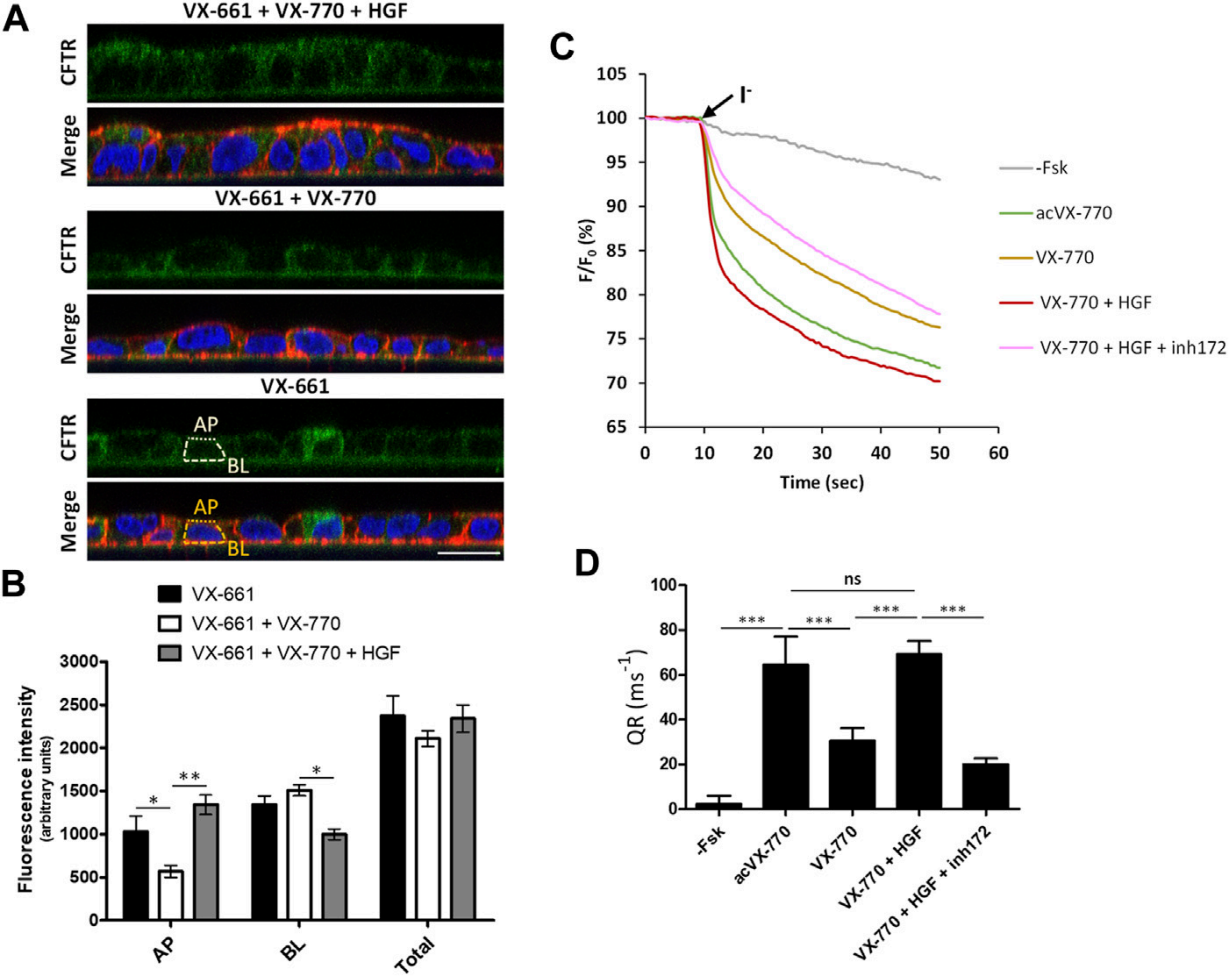
Co-treatment with HGF improves the apical abundance and function of rescued F508del-CFTR after prolonged exposure to VX-661+Vx-770 modulator combination. **(A)** Immunofluorescence staining of polarized F508del-CFTR CFBE cells treated for 15 days with either VX-661 (3 μM) alone, VX-661 plus VX-770 (1 μM), or VX-661+VX-770 and 50 ng/ml HGF, were stained with anti-CFTR/Alexa 488 (green), phalloidin-TRITC (red) and DAPI (blue), and analyzed by confocal microscopy as in Figure 2A. **(B)** Plotted are means ± SEM of AP, BL and Total (BL + AP) signal intensities from at least 25 cells analyzed in each of three independent experiments. Two-way ANOVA identified significant variation in CFTR’s subcellular localization among treatments (*F* = 61.01, *p* < 0.0001). Bonferroni posttests were used to compare treatments at the different subcellular localizations. *p < 0.05; **p < 0.01. **(C)** Representative traces of fluorescence decay on iodide influx assays of polarized HS-YFP/F508del-CFTR CFBE cells treated for 15 days with 3 μM of VX-661, alone or together with VX-770 (1 μM), or VX-770 and HGF (50 ng/ml). Cells were then stimulated with either DMSO (- Fsk), 5 μM forskolin, or 5 μM forskolin and 10 μM VX-770 (acVX-770) for 30 min, in the presence or absence of 25 μM CFTR inhibitor 172 (inh172). Fluorescence decay was recorded and analyzed as in Figure 2C. **(D)** Fluorescence decay rates (QR) were calculated as described in Figure 2D. Data are means ± SEM of five independent assays. Statistical significance between treatments was assessed using one-way ANOVA (*F* = 229.5, *p* < 0.0001) followed by Tukey’s posttests (***p < 0.001, ns = not significant).

However, as mentioned above, F508del correctors are usually administrated in combination with potentiator drugs, namely VX-770, to improve the rescued channels’ impaired gating (Meoli et al., 2021). We found that, as was described for VX-809 (Cholon et al., 2014; Veit et al., 2014; Matos et al., 2018), chronic (15 days) co-exposure to 1 µM VX-770 significantly (*p* < 0.01) reduces VX-661-rescued CFTR in F508del-expressing cells (Figures 3A,B). However, we found that co-administration of HGF restored rF508del abundance in VX-661+VX-770-treated cells to levels equivalent to cells treated with VX-661 alone (Figures 3A,B). This is consistent with the described role of HGF in improving the stability of rescued F508del-CFTR at the cells’ membrane (Moniz et al., 2013; Matos et al., 2018). Indeed, analysis of CFTR subcellular distribution in cells treated in these conditions clearly showed a significant decrease in apical localization of VX-661-rescued F508del-CFTR upon prolonged co-treatment with VX-770, which was completely reversed, and even favored, in the presence of HGF (Figures 4A,B). Importantly, iodide influx assays showed that this restoration of apical localization was sufficient to recover CFTR-mediated ion transport in chronic VX-661+VX-770 + HGF co-treated cells to levels equivalent to those of cells treated with VX-661 alone and acutely stimulated with 10 µM of VX-770 for 30 min (Figures 4C,D).

## Conclusion

Taken together, our results suggest that, as proposed for VX-809-based combination therapies (Matos et al., 2018), HGF co-treatment would also favor therapeutic regimens employing the chronic co-administration of VX-661 and VX-770, namely Symdeko^®^/Symkevi^®^ (United States and Europe commercial designations, respectively), currently approved for patients aged ≥6 years, homozygous for the F508del mutation or heterozygous for the F508del mutation and one of several residual function mutations (Meoli et al., 2021). While the physiologic significance of our findings is limited by the use of *in vitro* models, these should stimulate the CF scientific community to further address the potential gains of adding HGF to current CFTR modulator combinational therapies, namely by using currently available *in vivo* and *ex vivo* (patient-derived tissues and organoids) models. Supportive of a potential application of HGF in the CF setting, several *in vivo* studies indicated that HGF administration can mitigate the effects of acute and chronic lung injuries (Panganiban and Day, 2011), having beneficial effects both at the initial and late stages of lung disease (Yaekashiwa et al., 1997; Panganiban and Day, 2011). Moreover, HGF was shown to inhibit amiloride-sensitive epithelia Na^+^ channel (ENaC) function in CF airway epithelium (Shen, Widdicomb, and Mrsny 1999), suggesting that its administration could also be beneficial to reduce the abnormally high activity of ENaC observed in CF airway cells. In future studies, it will be interesting to ascertain if HGF can also improve the activity of the very recently approved triple combination of VX-661+VX-770 with VX-445, which has already shown better clinical responses (Meoli et al., 2021).

## Data Availability

The original contributions presented in the study are included in the article/Supplementary Material, further inquiries can be directed to the corresponding author.
